# Annotations

**Published:** 1909-08-28

**Authors:** 


### ANNOTATIONS.

### Physical Exercises in Schools.

During the past week the Board of Education has published a revised syllabus of physical exercises for the use of local educational authorities and teachers of elementary schools. This is an interesting memorandum, and a hopeful sign of the fact that the powers that be are amenable to suggestions from outside, even in a sphere where hitherto outside advice has been received with some scorn and much opposition. Reformers in education have always had a struggle in making their efforts for the improvement of existing measures felt, and none have had a harder battle to fight than those who insisted that in modern times more attention is being paid to the mind than to the body, and that sanity in intellect is to be greatly helped and encouraged by sanity of body. The present memorandum must make pleasant reading for these supporters of physical education. The Board of Education not only comes briskly into line with them, but it gives the full weight of its official authority to the suggestions that have been made and which must have the cordial approval of all medical men. Every school inspector, whether medical or otherwise, knows the regrettable lack of bodily stamina that is shown by 50 per cent. of pupils in elementary public schools. In private and higher-grade schools the pupils compare much more favourably with those, for example, to be found in German institutions where physical training is made as much of as purely intellectual teaching, but in elementary schools the physical standard is lamentably low. It is to be hoped that the suggestions now officially brought to the notice of teachers by the Board will be carefully considered, mastered, and given a trial by those whose care it is to watch over the younger generation. Much can be done by efficiently and solidly supporting the Board in this matter. A great deal of the physical unfitness to be seen in our schools is a result of ignorance, not of inherent disease or actual pathological change, and is therefore remediable. In rare cases only it may be the province of the medical inspector to deal with it: in general the teachers can do much to bring about a better state of affairs by paying attention, in the way suggested in the recently issued memorandum, to the physical culture of the pupils under their charge. To professional readers the importance of such physical training need not be pointed out, but to the laity it is a matter that cannot be too often and too authoritatively discussed.

### Forty Years of Hospital Work.

During the past forty years much progress and many improvements have been made in hospitals and hospital work all over the world. In England the changes that have been effected during these decades are striking in the extreme, and it would be both interesting and instructive for anyone conversant with the conditions of institutional life forty years ago to give his impressions of English hospitals as he finds them to-day. It is over forty years since Sir Henry Burdett made his first inspection of hospitals. At that time asepsis was unknown as a routine practice, the mortality after operations was from 33 to 40 per cent., that of puerperal fever scarcely much lower; there were no trained nurses, nor was there any system for training and instructing women engaged as attendants upon the sick. Neither the general hygiene nor the admiinistration of hospitals was perfect. To-day all this nas been changed. Pasteur's discoveries, Lister's application to surgery of the theories which Semmelweis had already enunciated in midwifery, greater attention to details of construction in hospital building, and to elementary essentials of hygiene, have been principally instrumental in effecting this wonderful change. Having been actively engaged in hospital work all through this time of transition, reform, and rearrangement, Sir Henry's observations on what has been accomplished will possess a special interest for the public, the profession, and all who take an interest in hospital work. He will commence a tour of inspection of the principal English hospitals this week, visiting every institution of importance and carefully contrasting and comparing it with its forerunners. We hope to publish the results of his tour in the form of special reports in the new series of The Hospital. The first report will appear in the first issue in October next.

### Sleeping Sickness.

The *Sleeping Sickness Bureau Bulletin*, No. 9, 1909, edited by Dr. Bagshawe, contains an account of some recent researches into the ætiology and treatment of sleeping sickness. Kleine has lately shown that tsetse flies, after sucking up trypanosomes from the blood of an infected animal, remain harmless for 17 days or so, but that after that period of time they are capable of transmitting infection. This briefly means that the parasites (the trypanosomes) have undergone some development, probably sexual, in the stomach of the insect, so that after the necessary time is over they or their young forms can find their way back, *viâ* the proboscis, into new hosts. The author (Kleine) now continues his work and studies the forms of the trypanosomes seen in the infected flies. These are very varied, and, as far as one can judge, might quite easily represent totally different species. As regards treatment of trypanosomiasis, the same lines are still in force, with a tendency to replace the older drugs by newer and less toxic ones. Atoxyl, owing to the toxic symptoms that have followed its use, is now hardly ever given; its place is taken by soamin, its English equivalent, or by arsacetin, an improved compound. Newer still is Ehrlich's arseno-phenyl-glycin, a drug from which great results are expected. This was recently tried for the first time in human therapeutics by Alb, who gave it to patients suffering from general paralysis. No toxic signs appeared, and no unusual symptoms developed, and this being so, it will probably be quickly tried in human trypanosomiasis.

